# Laparoscopic management of pathologic gastroesophageal reflux after sleeve gastrectomy using the magnetic sphincter augmentation (MSA) device—a Video Vignette

**DOI:** 10.1007/s11695-022-06007-x

**Published:** 2022-03-15

**Authors:** Davide Bona, Marco Antonio Zappa, Valerio Panizzo, Andrea Sozzi, Caterina Lastraioli, Francesca Lombardo, Cristina Ogliari, Alberto Aiolfi

**Affiliations:** 1 Department of Biomedical Science for Health, Division of General Surgery, Istitituto Clinico Sant’Ambrogio, University of Milan, Via Luigi Giuseppe Faravelli, 16, 20149 Milan, Italy; 2 UOC Di Chirurgia Generale, ASST Fatebenefratelli Sacco, Milano, Italy

**Keywords:** Laparoscopic Sleeve Gastrectomy, GERD, Magnetic sphincter augmentation device

## Abstract

**Purpose:**

The development of gastroesophageal reflux disease (GERD) has been shown to be not infrequent after laparoscopic sleeve gastrectomy (LSG). Management may vary from medical therapy to Roux-en-Y gastric bypass (RYGB) conversion. Magnetic sphincter augmentation (MSA) device has been shown to be a promising option with excellent results. The purpose of this video was to demonstrate the laparoscopic management of post-LSG GERD with MSA device implant.

**Materials and Methods:**

An intraoperative video has been edited to demonstrate the MSA device placement after LSG for the treatment of pathologic GERD.

**Results:**

The procedure started with the lysis of the perigastric adhesions to free the distal esophagus circumferentially. The posterior vagus nerve was identified, and a small window was created between the posterior esophageal wall anteriorly and the vagus nerve posteriorly. A hiatoplasty was performed using two non-resorbable interrupted 2.0 Prolene® sutures. The system’s sizer was placed to measure the junctional circumference. A 15-mm MSA device was implanted.

**Conclusion:**

MSA device placement seems technically feasible and safe with promising results in term of improved LES resting pressure and esophageal acid exposure. While future studies are necessary to corroborate these preliminary indications, MSA device may possibly become a valid option in surgeon armamentarium.

**Supplementary Information:**

The online version contains supplementary material available at 10.1007/s11695-022-06007-x.

## Introduction

Laparoscopic sleeve gastrectomy (LSG) has gained progressive worldwide acceptance [1]. The development of “de novo” or the worsening of latent gastroesophageal reflux disease (GERD) has been shown to be not infrequent [2–4]. Management of post-LSG GERD may vary from proton pump inhibitors (PPI) therapy to Roux-en-Y gastric bypass (RYGB) conversion [5, 6]. Magnetic sphincter augmentation (MSA) device has been shown to be a promising option in non-obese patients with GERD with excellent results in term of esophageal acid exposure normalization, low complication rates and quality of life improvement [7]. As the number of patients with post-LSG GERD will grow in the future because the increasing number of performed procedures, MSA device implant may constitute an attractive option in surgeon armamentarium.

## Purpose

The purpose of this video was to describe the management of GERD in a 45-year-old female patient (Body Mass Index: 27.7 kg/m^2^). The patient was referred to our institution for heartburn and regurgitation (7 years after LSG). The preoperative Gastroesophageal Reflux Disease-Health Related Quality of Life (GERD-HRQL) was 37. The upper endoscopy showed the presence of a 2-cm hiatal hernia with grade A esophagitis. The high-resolution manometry (HRM) showed hypotensive lower esophageal sphincter (LES) (7 mmHg) with normal esophageal body peristalsis. The 24 pH-impedance study showed pathologic distal esophageal acid exposure (DeMeester score: 68.7).

## Methods

An intraoperative video has been edited to demonstrate the feasibility of MSA device placement after LSG. Written informed consent was obtained from the patient.

## Results

The procedure started with the section of the perigastric adhesions to free the distal esophagus circumferentially. Esophageal dissection was completed to obtain 3 cm of distal esophagus without tension in the abdomen. The posterior vagus nerve was identified, and a small window was created between the posterior esophageal wall and the neural branch. Cruroplasty was performed using two non-resorbable interrupted 2.0 Prolene® sutures. The system’s sizer was placed to measure the junctional circumference. A 15-mm MSA device was chosen. The operative time was 45 min. The postoperative course was uneventful. At 25-month follow-up, the patient was asymptomatic with a normal distal esophageal acid exposure (DeMeester score: 9.7) and LES resting pressure restoration (16 mmHg).

## Discussion

The prevalence of GERD after LSG has been reported up to 22% [8]. Several factors have been indicated as possible causes such as dilation in the proximal pouch, LES weakening, increasing number of transient LES relaxations (TLESR) and hiatus hernia [9]. The challenging management of such patients has been through medical PPI treatment or conversion to RYGB. The use of MSA for the treatment of post-LSG GERD has been described in previous studies [10–12]. However, the narrow sample size and limited follow-up limited the validity of such papers. In the present study, we describe the medium-term outcomes after MSA device implant with LES resting pressure increase, distal esophageal acid exposure restoration and improved quality of life (GERD-HRQL: 6) [13].

Despite its rarity, MSA erosion has been described as potential MSA device implant drawback [14]. MSA device size mismatch, infection, as well as patient-related factors such as connective tissue disorders, steroids use and immunosuppression have been identified as potential risk factors for erosion [15]. To prevent size mismatch, we suggest to ratchet down the esophageal sizer until it releases from encircling the esophagus; two sizes above the release size are appropriate to avoid undersizing. In the present case, the decision for MSA placement was made because the patient reached a reasonable BMI, while conversion to RYGB would have introduced unnecessary operation-related risks and potential malabsorption. Because its standardization, reproducibility and promising outcomes MSA device implant may be considered in patients with pathologic GERD, hypotensive LES, normal esophageal peristalsis and acceptable postoperative weight loss.

## Conclusion

MSA device placement seems technically feasible and safe with promising results. While future and large scale studies are mandatory to corroborate these preliminary indications, MSA device may potentially become an attractive and viable option in the surgeon armamentarium. Specific indications and universally accepted guidelines are required to identify patients that might benefit from this approach.

## Supplementary Information

Below is the link to the electronic supplementary material.

Supplementary file1 (MP4 275143 kb)
